# Zoning of Ecological Restoration in the Qilian Mountain Area, China

**DOI:** 10.3390/ijerph182312417

**Published:** 2021-11-25

**Authors:** Lin Liu, Wei Song, Yanjie Zhang, Ze Han, Han Li, Dazhi Yang, Zhanyun Wang, Qiang Huang

**Affiliations:** 1 School of Land Science and Space Planning, Hebei GEO University, Shijiazhuang 050031, China; liulin7801@126.com; 2 Key Laboratory of Land Surface Pattern and Simulation, Institute of Geographic Sciences and Natural Resources Research, Chinese Academy of Sciences, Beijing 100101, China; zhangyanjie_cdlg@yeah.net (Y.Z.); hanze1125@163.com (Z.H.); lih.19s@igsnrr.ac.cn (H.L.); yangdazhi@igsnrr.ac.cn (D.Y.); wangzhanyunwzy@163.com (Z.W.); 3 College of Resources and Environment, University of Chinese Academy of Sciences, Beijing 100049, China; qiang12031@foxmail.com; 4 Institute of Mountain Hazards and Environment, Chinese Academy of Sciences and Ministry of Water Conservancy, Chengdu 610041, China; 5 State Key Laboratory of Resources and Environmental Information System, Institute of Geographic Sciences and Natural Resources Research, Chinese Academy of Sciences, Beijing 100101, China

**Keywords:** land use zoning, ecosystem system, ecological restoration, Qilian Mountains, China

## Abstract

Ecosystem restoration has been widely concerned with the damage and degradation of ecosystems worldwide. Scientific and reasonable formulations of ecological restoration zoning is the basis for the formulation of an ecological restoration plan. In this study, a restoration zoning index system was proposed to comprehensively consider the ecological problems of ecosystems. The linear weighted function method was used to construct the ecological restoration index (ERI) as an important index of zoning. The research showed that: (1) the ecological restoration zones of the Qilian Mountains can be divided into eight basins, namely the headwaters of the Datong River Basin, the Danghe-Dahaerteng River Basin, the northern confluence area of the Qinghai Lake, the upper Shule River to middle Heihe River, the Oasis Agricultural Area in the northern foothills of the Qilian Mountain, the Huangshui Basin Valley, Aksay (corridor region of the western Hexi Basin), and the northeastern Tsaidam Basin; (2) the restoration index of the eight ecological restoration zones of the Qilian Mountains was between 0.34–0.8, with an average of 0.61 (the smaller the index, the more prominent the comprehensive ecological problem representing the regional mountains, rivers, forests, cultivated lands, lakes, and grasslands, and thus the greater the need to implement comprehensive ecological protection and restoration projects); and (3) the ecological problems of different ecological zones are frequently numerous, and often show the phenomenon of multiple overlapping ecological problems in the same zone.

## 1. Introduction

Ecosystems as a whole connect biological organisms and the inorganic environment through a variety of ecological functions, which feature comprehensive, holistic, and systematic characteristics. The unreasonable use of natural resources has caused many ecological problems, such as soil erosion, land desertification, and the degradation of forest and grass vegetation. Many ecological problems have caused widespread concern for the restoration of ecosystems. For the formulation of ecological restoration plans in a scientific and reasonable manner, it is necessary to comprehensively analyze the problems existing in ecosystems and identify the key restoration areas. From this point of view, the demarcation of ecological restoration zoning is of great significance.

The earliest studies of ecosystem zoning can be traced back to the zoning studies of natural ecosystems. In the early 19th century, the isothermal graph of the German geographer A.V. Humboldt marked the beginning of the study of natural ecological zoning [1]. On this basis, the concept of ecological zones and their divisions, as proposed by Merriam, became the prototype of ecological divisions [2]. Since then, ecologists have continued to research the principles of ecological divisions. Ecological zoning during this period mainly considered the natural factors affecting the ecosystem, and the functional factors of the ecosystem itself were less prioritized. In the 1980s, ecosystem functional divisions were created, which were widely used for large-scale regional natural resource management by the Environmental Cooperation Commission [3], the World Wildlife Fund [4], and the Food and Agriculture Organization of the United Nations. In the initial stage of ecosystem functional zoning, the research areas were mainly considered on a large or medium scale [5,6], as in the case of the construction of large-scale ecosystem zoning systems in Europe, North America, and Africa [7,8]. Since then, researchers have focused more on the zoning of single ecosystems, especially in water environment ecosystems [9,10,11].

In recent years, the deterioration of ecosystems has become an important factor restricting socio-economic development [12,13], and the study of the restoration zoning of ecosystems has received increasing attention. For example, Li et al. [14] took China’s National Ocean Park, in the Haizhou Bay National Ocean Park, as their research area. Based on the evaluation of the ecological environment of the Haizhou Bay Reserve, the ecosystem health of the island’s terrestrial ecosystem, intertidal ecosystem, and shallow sea ecosystem were assessed, and the ecosystem restoration zones were divided according to a vulnerability assessment. Yang et al. [15], using the ecological compensation zoning index of cultivated lands and the financial payment model of cultivated lands’ ecology, divided the cultivated lands’ ecological compensation areas in Wuhan. Wang et al. [16], taking the Tarbagatay Basin in the Xinjiang province as an example, constructed an evaluation index system from three aspects of the suitability of ecological protection, urban development, and agricultural production, and divided the ecological, agricultural, and urban space regions of the Tarbagatay Basin.

For the zoning research method, the current commonly used methods mainly include main component analysis, index partition, empirical partition, graph cascade adding, and cluster partition. In recent years, due to the rapid development of computer and remote sensing technologies, the means of ecological zoning research has become more and more advanced. The quantity of regional research is increasing due to software and technologies such as ArcGIS, SPSS, Matlab, and neural networks. For example, Wang et al. [17], using the Sunan Yugu Autonomous County in the Qilian Mountain District as an example, discussed ecological space demarcation and zoning methods based on the ArcGIS platform. Some scholars [18,19] have also carried out zoning based on ArcGIS technologies based on the analysis of regional environmental statuses and spatial variations in ecological sensitivity. In another example, Jing et al. [20] proposed a regional-scale ecological protection zone division method based on an improved artificial bee colony.

Overall, the zoning of the ecosystem underwent three stages: natural-element-based zoning, functional-based zoning, and ecological-restoration-oriented zoning. Given the urgency for ecosystem restoration, research on ecosystem restoration zoning has received increasing attention in recent years. However, in this study, there were some problems both in the concept of zoning and its indicators. For example, how ecosystem zoning changes from the partitioning of a single ecosystem component into an integrated ecosystem restoration partition is worthy of attention [21].

In summary, research on ecosystem zoning has entered a new era in which more attention is being given to ecosystem restoration zoning. However, due to the inadequacy of zoning concepts and index system construction, there are still great challenges related to ecosystem restoration zoning. In light of this, taking the Qilian Mountains of China as an example, the ecological restoration zone of the Qilian Mountains was developed based on the concept of the integrated protection and restoration of mountains, rivers, forests, cultivated lands, lakes, and grasslands. Specifically, the research goals of this paper were to: (1) reveal the land use restoration of the Qilian Mountains in recent years; (2) put forth a new set of ecological restoration zone index systems based on the concept of the life communities of mountains, rivers, forests, cultivated lands, lakes, and grasslands; and (3) to delimit the ecological restoration zones of the Qilian Mountains scientifically and reasonably. The paper is structured as follows:

- In Section 2, we introduce the study area and data sources.
- In Section 3, we introduce the methods used for ecological restoration zoning.
- In Section 4, we document the key areas of ecological restoration and their ecological problems.
- The limitations and future research prospects are discussed in Section 5.
- The conclusions are then presented in Section 6.

## 2. Study Area and Data Sources

### 2.1. Study Area

**Figure 1:**
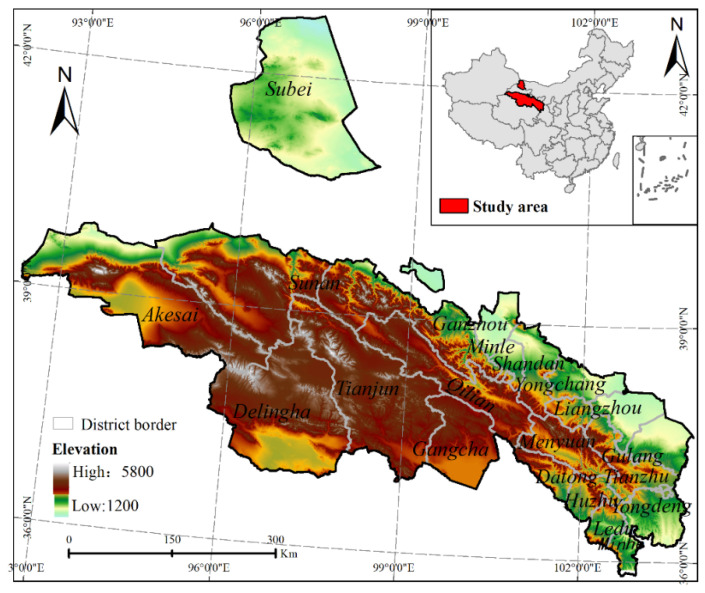
Geographic location of the Qilian Mountains in China.

The Qilian Mountain area is one of the main mountainous provinces in China (Figure 1), situated in the northeast of the Qinghai Province and the western border of the Gansu Province (northeast of the Qinghai–Tibet Plateau) between 94°20′–103° E, 36–40° N, with a total area of 237,000 km^2^. The annual average temperature in the study area is 1.4 °C below zero to 9.6 °C, and the total amount of solar radiation is 5916–15,000 MJ/m^2^. The annual average precipitation is between 0–700 mm. The Qilian Mountain area consists of a number of northwest–southeast parallel mountains and wide valleys. The mountains mainly include the Daxue Mountain, Tuolai Mountain, Tuolai South Mountain, Yema South Mountain, Shule South Mountain, Danghe South Mountain, Tuergen Daban Mountain, Chai Damu Mountain, and Zongwulong Mountain. The Qilian Mountains have an average elevation of 4000–4500 m, and many peaks are over 5000 m.

### 2.2. Data Sources

The data used in this paper mainly include soil data, land use data, desertification data, cultivated land quality data, and statistical data. Among them, the soil organic matter content and soil texture data were derived from the Harmonised World Soil Database (HWSD) [22]. Land use data come from remote sensing monitoring data of land uses in China in 2015 [23]. In this monitoring data, land use types include six primary types and twenty-four secondary types. The desertification data were obtained from the 1:100,000 Chinese Desert Gobi Distribution Map for 2000 [24]. The cultivated land quality data come from a 1:1 million land resource map [25]. The assessment of the change status of forest grassland vegetation is based on the 16-day synthetic NDVI products (MYD13A2 and MOD13A2) of MODIS from 2001–2015 with a spatial resolution of 1000 m [26]. The animal husbandry statistics at the county level come from the provincial statistical yearbooks, and the corresponding rural income per capita income come from the Gansu Development Yearbook for 2015 [27] and the Qinghai Statistical Yearbook for 2015 [28]. The mine distribution density was obtained from a large network crawler data search. Precipitation data were derived from the China Ground Climate Data Daily Value Dataset (V3.0) [29], and the spatial distribution was obtained by interpolation.

## 3. Research Methods

### 3.1. Index System Construction

The ecological development, overall protection, and comprehensive governance of all ecological and environmental elements are required for the ecological restoration of the “life community” of mountains, rivers, forests, cultivated lands, lakes, and grasslands. The demarcation of the ecological restoration zones of the Qilian Mountains aims to consider the comprehensive ecological problems related to the ecological factors of mountains, rivers, forests, cultivated lands, lakes, and grasslands in the Qilian Mountains. These comprehensive ecological problems include soil erosion, forest and grass quality degradation, cultivated land quality degradation, and water and soil erosion caused by mining, etc. We constructed the regional evaluation index system of ecological restoration in the Qilian Mountains according to the principles of scientific nature, systems, correlations, and operability; eight evaluation indicators were considered, including the amount of soil conservation, mine distribution densities, rainstorm days, annual precipitation, interannual change rates of forestry area vegetation, agricultural production potential, interannual change rates of grassland vegetation, and rural income per capita (Table 1).

Among them, soil erosion and mining are the main ecological problems related to mountain elements. The diagnostic index of the ecological problems of soil erosion is soil conservation [30], and the diagnostic index of ecological problems in mining is the mine distribution density [31]. The imbalance of extreme precipitation and precipitation distribution is an ecological problem related to water [32]. The diagnostic index of extreme precipitation is represented by rainstorm days, and the diagnostic index of unbalanced precipitation distribution is the spatialized annual precipitation. The degradation of forested areas is an ecological problem related to forests, and its diagnostic index is the interannual change rate of forest land vegetation [33]. The quality of cultivated lands is an ecological problem related to cultivated lands, and its diagnostic index is its agricultural production potential [34]. Grassland degradation is an ecological problem related to grasslands, and its diagnosis index is the interannual change rate of grassland vegetation [33]. Poverty status is a human-related problem. Although poverty may not be an ecological problem, poverty is closely related to the emergence of ecological problems. The diagnostic index of poverty status selection is the rural per capita income [35]. The specific calculation process of indicators is shown in Appendix A.

### 3.2. Ecological Restoration Index (ERI) of the Qilian Mountains

We used the linear weighted function method to construct the regional ecological restoration index (ERI) and analyze the spatial differences by using the spatial clustering and grouping method. The specific steps are as follows:

First, with different units, meanings, and contents, there are differences in data dimensions and trend directions. Therefore, each index must be standardized. The formula was as follows [36]:

(1) $$\left\{ \begin{array}{l} Positive\;index:x_{ij}^{\prime }=\frac{x_{ij}-x_{j}^{\min}}{x_{j}^{\max}-x_{j}^{\min}}\\ Negative\;index:x_{ij}^{\prime }=\frac{x_{j}^{\max}-x_{ij}}{x_{j}^{\max}-x_{j}^{\min}} \end{array}\right.$$

Second, according to the number of ecological factors of mountains, rivers, forests, cultivated lands, lakes, grasslands, and people involved in various ecological indicators, the weight of various ecological indicators was determined. In other words, if this index involved only one ecological problem related to mountains, rivers, forests, cultivated lands, lakes, and grasslands, the value was 1; if two ecological problems are involved, the value was 2; and so on. Then, the score of each indicator was expressed as a percentage (Table 2) representing the indicator weight.

Finally, based on the constructed ERI system and weight, the linear weighted function method was used to measure the ERI. The comprehensive situation of the ecological problems of regional mountains, rivers, forests, cultivated lands, lakes, and grasslands can be reflected through this indicator. The overall rule is that the smaller the ERI, the more prominent the comprehensive ecological problems representing mountains, rivers, forests, cultivated lands, lakes, and grasslands, and the more necessary it is to carry out comprehensive ecological restoration projects. The calculation formula was as follows [37]:

(2) $$ERI=\sum_{i=1}^{n}w_{i}\times E_{i}$$

where “*E_i_*” is the value of the *i*-th ecological indicator and “*w_i_*” is the weight of the *i*-th ecological indicator.

### 3.3. Division of Basic Evaluation Units Based on River Basin Division

From the perspective of systematic restoration, we extracted small basins in the study area as the basic units for the evaluation of ecological restoration zoning. Basin extraction, or catchment extraction, is the joint determination of its spatial scope based on the river’s flow direction and outlet. From a hydrology and geography perspective, its region must correspond to that of the river. Therefore, rivers must be designated before the watershed extraction. River data can be extracted from digital elevation model (DEM) data or converted from existing vector rivers. We used a slope runoff simulation algorithm to realize automatic water system extraction and river basin segmentation. The main steps were as follows:

First, we determined the direction of the water flow of the grid unit. After preprocessing the DEM data, the flow direction was calculated. We then extracted the depression, analyzed the threshold of the depression, and set the threshold to fill the DEM data. This step was repeated until all existing depressions in the DEM were eliminated to lay the basis for the hydrological analysis.

Second, the drainage basin was calculated, and the flow accumulation matrix was determined by the flow direction. Next, the upstream catchment area of each grid unit was obtained. Then, the appropriate confluence threshold was determined. The confluence threshold value needed to be measured repeatedly, and the threshold value was inversely proportional to the number of river basins. The water exchange area extraction was conducted based on the flow direction. After determining the basin outlet grid, all grids to the outlet could be searched according to the flow direction matrix to obtain the basin boundary and that of the sub-basin in order for basin segmentation to be realized.

**Figure 2:**
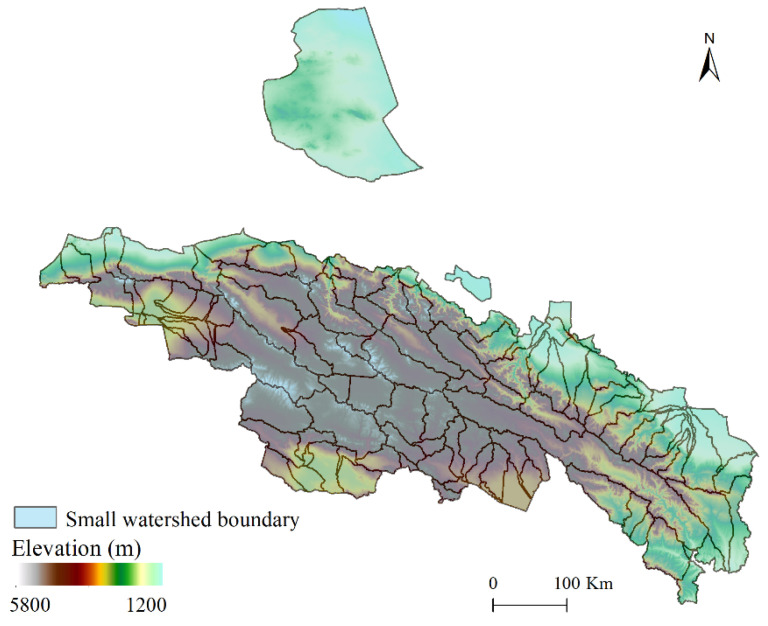
Small watershed division of the Qilian Mountains.

Finally, the Qilian Mountain area was divided into 108 small river basins (Figure 2).

## 4. Results

### 4.1. Land Use Change in the Qilian Mountains from 1990–2015

**Figure 3:**
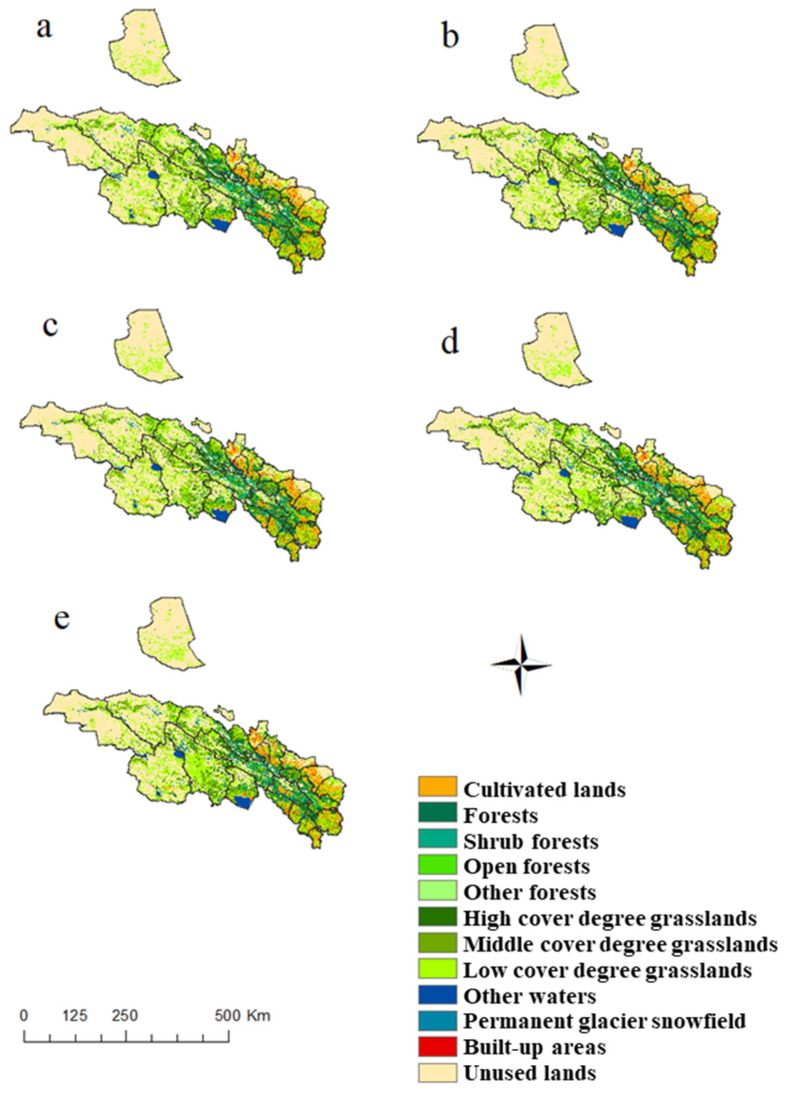
Types of land use in the Qilian Mountains in 1990 (**a**), 2000 (**b**), 2005 (**c**), 2010 (**d**), and 2015 (**e**).

The cultivated lands in the Qilian Mountains are mainly distributed between Ganzhou, Minle, Yongchang, Shandan, and Gulang in the northeast and Huzhu, Ledu, and Minle in the southeast, and most of them are dry lands (Figure 3). The forestry areas are mainly distributed in Qilian and Menyuan in the central region. Grasslands and unused lands collectively account for approximately 80% of the study area, mainly at high elevations. Among them, the grasslands are mainly distributed in the center of the study area, while the unused lands are distributed in the center and the northeast. The water areas comprise the smallest type in the research area, featuring blocks distributed in the middle and south of the study area. The built-up areas are scattered in the eastern and southeastern regions, which also correspond to the distribution areas of cultivated lands.

**Figure 4:**
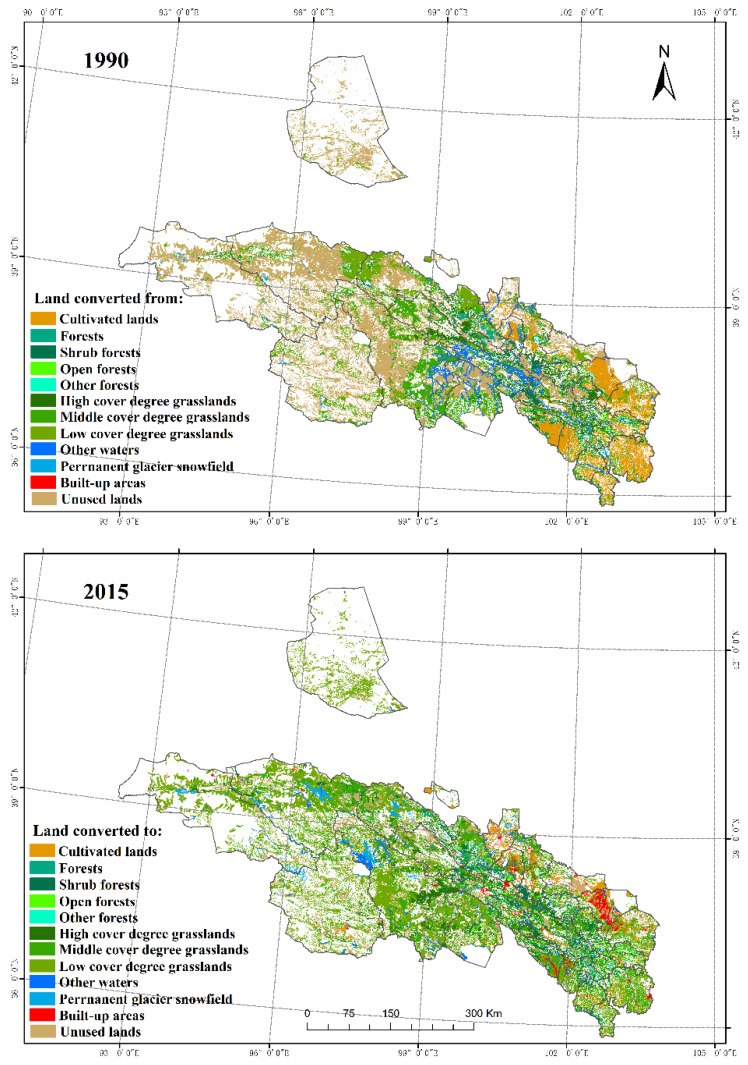
Land use transfer matrix in the Qilian Mountains from 1990 to 2015.

From 1990–2015, the cultivated lands, grasslands, and built-up areas of the Qilian Mountains generally increased (Figure 4), while forestry areas, water bodies, and unused lands generally declined. The cultivated lands in the Qilian Mountains increased by 0.31% from 1990 (5.8%) to 2015 (6.11%). Of these cultivated lands, 0.79% were converted into other land use types, and the proportion of converted to cultivated lands was 1.11%. In terms of spatial change, the area of cultivated lands is mainly part of Ganzhou and Sunan; meanwhile, in the southeast of Sunan, Menyuan, Tianzhu, and Yongdeng, a large proportion of the cultivated lands has been transformed into grasslands and built-up areas. As with cultivated lands, the area of grasslands has also shown an increasing trend over the years, increasing by 1.28% from 1990 (36.85%) to 2015 (38.13%). The Qilian Mountain area is mainly dominated by medium- and low-cover grasslands. From 1990–2015, the conversion of high-, medium-, and low-complexity grasslands to other land use types was 0.71%, 2.12%, and 2.72%, respectively. The proportions of conversion to high-, medium-, and low-complexity grasslands were 0.015%, 1.05%, and 1.84%, respectively. Spatially, the grasslands increased in the northeast and southeast of the Qilian Mountains, and the main reason for the decrease in grasslands was that the grasslands were transformed into unused lands; however, because the proportion was small, the spatial performance was not obvious. 

The built-up areas increased from 0.36% in 1990 to 0.54% in 2015, with an increase of 0.18%. In terms of spatial change, in the southeast region of the research area, the built-up areas showed a large increasing trend. In contrast, the forestry areas decreased from 6.12% in 1990 to 6.10% in 2015, with a decrease of 0.02%. Of this latest percentage, the forestry areas, scrubs, sparse, and other land use types made up 0.18%, 0.48%, 0.22%, and 0.01% of the total, respectively. The proportion of lands converted from other lands into forested, shrub lands, open woodlands, and other wooded lands accounted for 0.19%, 0.46%, 0.21%, and 0.00% of the total, respectively. Spatially, the areas with forests were basically converted into grasslands, so the areas where grasslands increased just happened to be the areas of forest reduction. The water area increased by 0.20%, from 1.75% in 1990 to 1.95% in 2015. Within these areas, the conversion from water to other land types was 0.17%, and 3.94% from other land types into water bodies. Due to the impact of global warming, the Qilian Mountain glaciers have experienced a large-scale retreat. The glaciers are in a state of material loss, generally receding and thinning. Coupled with the reduction in wetlands, the waters generally show a downward trend. Spatially, the areas with water decreased mainly in Tianjun, Gangcha, and Qilian. The proportion of unused lands decreased from 48.6% in 1990 to 46.38% in 2015, with a total decrease of 2.22%. The proportion of unused lands converted to other land use types was 4.43%, and that of other land use types converted to unused lands was 2.21%. The unused reductions were concentrated in large areas in the northeastern part of the study area, including in places such as Aksai, Tianjun, Delingha, and Sunan.

### 4.2. Key Areas of Ecological Restoration in the Qilian Mountains

**Figure 5:**
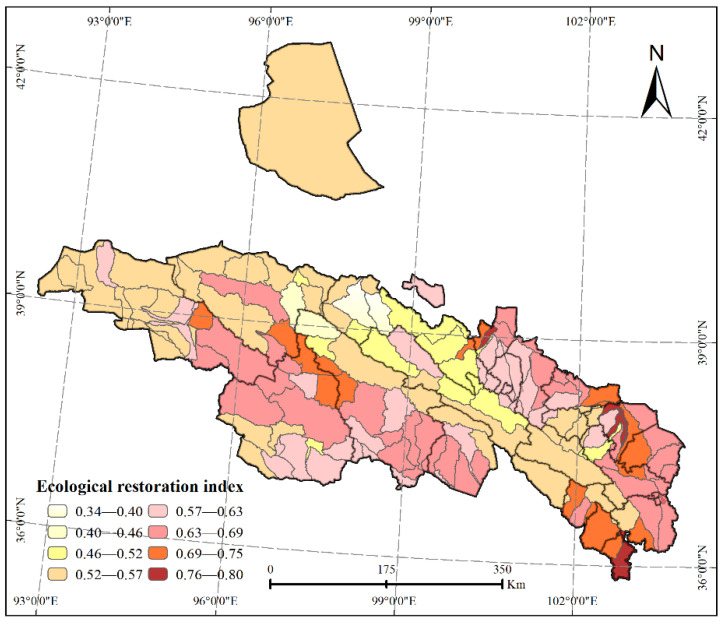
Ecological restoration index (ERI) of the Qilian Mountains.

Considering the eight factors of soil conservation, mine distribution density, extreme precipitation, annual precipitation, the annual change rates of areas with forestry vegetation and poverty level, quality of cultivated lands, and annual change rates of grassland vegetation and poverty level, and using the comprehensive evaluation system of ecological restoration, we calculated the comprehensive ERI of mountains, forests, cultivated land, lakes, and grasslands in the planning area (Figure 5). The index reflects the degree necessary to implement the comprehensive ecological restoration of mountains, rivers, forests, cultivated lands, lakes, and grasslands. The average of the ecological restoration indicator in the Qilian Mountains was 0.57, and the average in each basin ranged between 0.34 and 0.80. It can be seen that the ERI of the Qilian Mountain area showed a law of decreasing from the edges to inland. 

The ERI is an index that measures the key areas of ecological restoration. The greater the value, the greater the need to carry out ecological restoration work in the ecological area. It can be seen that the ERI in the southeast and southwest of the study area is relatively large. This means that there is an urgent need for ecological restoration in these areas.

### 4.3. Ecological Restoration Zoning of the Qilian Mountains

Due to the different natural resource endowments of the Qilian Mountains and the strong spatial heterogeneity of various ecological restoration indicators, the use of ecological restoration zones divided by the comprehensive ERI cannot further reflect the differences in the ecological restoration directions in each district, and it is not conducive to the appropriate measures. Therefore, this paper used an ArcGIS cluster analysis tool based on the calculation results of eight ecological restoration indicators and ERIs in each district, and divided them into two level divisions in accordance with the principle of natural division order.

**Figure 6:**
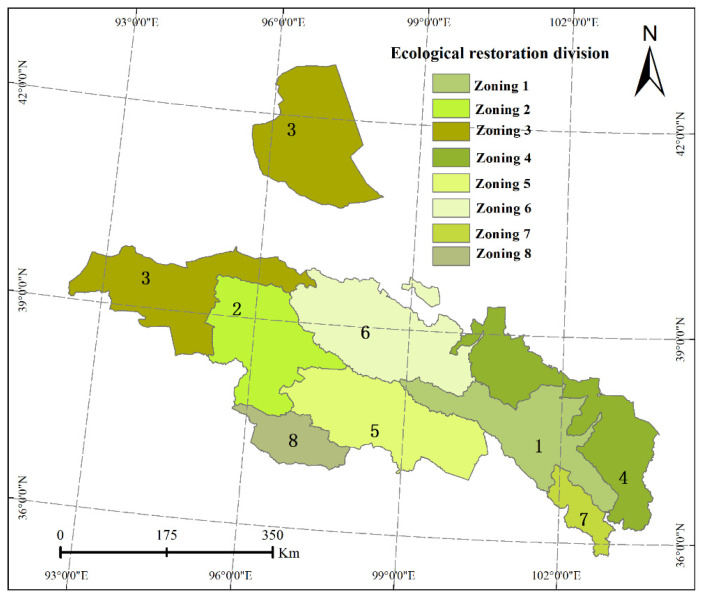
Ecological restoration zoning of the Qilian Mountains. Zoning 1 represents the headwaters of the Datong River Basin; Zoning 2 represents the Danghe-Dahaerteng River Basin; Zoning 3 represents Aksay (corridor region of the western Hexi Basin); Zoning 4 represents the Oasis Agricultural Area at the northern foothills of the Qilian Mountains; Zoning 5 represents the northern confluence area of the Qinghai Lake; Zoning 6 represents the upper Shule River to middle Heihe River; Zoning 7 represents the Huangshui Basin Valley; and Zoning 8 represents the northeastern Tsaidam Basin.

Finally, the Qilian Mountain area was divided into three primary districts and eight secondary districts. The first division included the forests and grassland water conservation areas, the ecological restoration areas of cultivated lands, and ecological control areas of deserted grassland (Table 3). The secondary ecological restoration zone included the headwaters of the Datong River Basin, the Danghe-Dahaerteng River Basin, the northern confluence area of the Qinghai Lake, the upper Shule River to middle Heihe River, the Oasis Agricultural Area at the northern foothills of the Qilian Mountains, the Huangshui Basin Valley, Aksay (corridor region of the western Hexi Basin), and the northeastern Tsaidam Basin (Table 3 and Figure 6).

### 4.4. Ecological Problems of Different Ecological Restoration Zones

**Figure 7:**
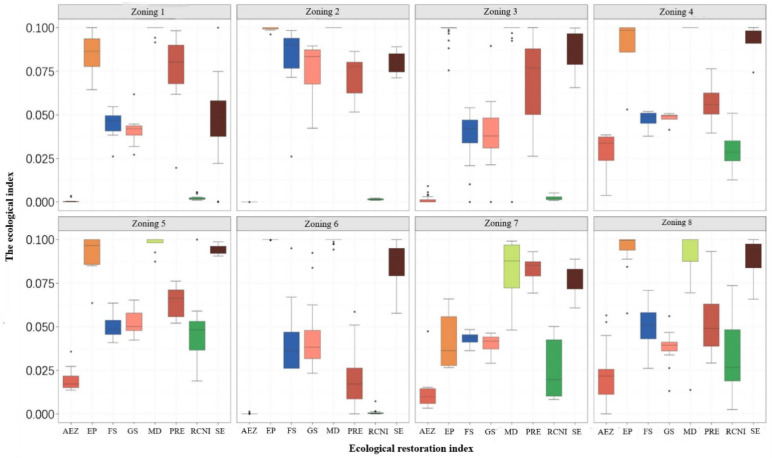
Ecological indicators of different ecological restoration zones of the Qilian Mountains. “AEZ” represents the agricultural production potential; “EP” represents extreme precipitation; “FS” represents the interannual change rate of the normalized difference vegetation index (NDVI) in forestry areas’ vegetation; “Rural per capita income” represents regional GDP; “GS” represents the NDVI interannual change rate of grassland vegetation; “MD” represents the mine distribution density; “PRE” represents the total annual precipitation; “SE” represents the soil erosion volume; Zoning 1 represents the headwaters of the Datong River Basin; Zoning 2 represents the Danghe-Dahaerteng River Basin; Zoning 3 represents Aksay (corridor region of the western Hexi Basin); Zoning 4 represents the Oasis Agricultural Area at the northern foothills of Qilian Mountain; Zoning 5 represents the northern confluence area of the Qinghai Lake; Zoning 6 represents the upper Shule River to middle Heihe River; Zoning 7 represents the Huangshui Basin Valley; and Zoning 8 represents the northeastern Tsaidam Basin.

Combined with the land use data, we further compared the differences of various ecological restoration indicators in different divisions (Figure 7) and discussed the main ecological problems of each ecological restoration division.

The headwaters of the Datong River Basin are located in the eastern section of the Qilian Mountains, and they are also the source of the Heihe, Shiyang River, and other river basins in the Qinghai and Gansu provinces. The main ecological functions of the area are water conservation and soil conservation. The district includes four counties: Qilian, Sunan, Menyuan, and Datong. The area covers 2.75 × 104 km^2^, accounting for 11.61% of the total regional area. Compared with other zones, the area is seriously affected by extreme precipitation, with greater extreme precipitation days and a large mine density (Figure 7). The interannual growth of forest vegetation is weak, and the production potential of cultivated land resources is very low.

The Danghe-Dahaerteng River Basin is located in the high mountains in the western section of the Qilian Mountains, including the three sub-basins of the Danghe River, Dahaerteng River, and Yema River. The counties comprising this area are Delingha, Subei, and Aksay. The main ecological functions of the area are water conservation, soil conservation, windbreaks, and sand fixation. The basin covers an area of 2.80 × 104 km^2^, accounting for 11.86% of the total regional area. The main ecological problem is the uneven distribution of water resources (Figure 7), showing a decline from east to west; such a low production potential further increases poverty.

The northern part of Aksay (corridor region of the western Hexi Basin) is the western section of the Hexi Corridor, with the Aksay Basin to its south. The two counties in this region Subei and Aksay. The main ecological functions of the area are windbreaks and sand fixation, as well as water–soil conservation. The area measures 6.11 × 10^4^ km^2^, accounting for 25.78% of the total regional area. The biggest natural problem of this zone compared to others is the sparse local precipitation and its uneven distribution (Figure 7). Droughts, less rain, and serious wind erosion have become the leading causes for its restricted ecological functions, and are also the source of other problems.

The Oasis Agricultural Area at the northern foothills of the Qilian Mountains is the core area of human activity and economic development in the Gansu Province. The main functions of the area are water and soil conservation, in addition to food production. The region covers an area of 3.43 × 104 km^2^, accounting for 14.45% of the total regional area. Compared with other subdivisions, the development of agriculture, animal husbandry, and mineral resources is intense; ecological problems such as overgrazing, vegetation degradation, soil erosion, and desertification are thus prominent, and the relationship between humans and the natural environment has gradually become unbalanced (Figure 7).

The northern confluence area of the Qinghai Lake is located in the middle of the Qilian Mountains, centering around the Qinghai Lake Basin, and reaching the Shule Nanshan Mountain in the north and the Qinghai Nanshan one in the south. The main functions of the area are water and soil conservation. The area is 3.28 × 104 km^2^, accounting for 13.84% of the total regional area. Compared with other divisions, the main ecological problems in the area include serious mountain soil erosion (Figure 7), as seen in the Buha River Basin and the southern foot of the Datong Mountain, thus often causing landslides and debris flows. Vegetation degradations in the northeast of the Qinghai Lake have also made the local microclimates unstable or even caused them to deteriorate. Finally, less cultivated land resources with a low production potential and poverty have destroyed the ecological environment.

The upper Shule River to middle Heihe River is located in the middle of the Qilian Mountains, south of the Shule Henan Mountain and north of the Hexi Corridor, including the upper reaches of the Shule River and the middle reaches of the Heihe River. The main functions of the area are water and soil conservation. The area covers 3.62 × 104 km^2^, accounting for 15.26% of the total regional area. Compared with other divisions, the soil development is poor, both the quantity and quality of cultivated lands are rather low, and the poverty index is high (Figure 7).

The Huangshui Basin Valley is located in the river valley area of the Huangshui River Basin, and is its central area of human activity and economic development. The main functions of the area are soil conservation and food supply. The region covers an area of 0.66 × 104 km^2^, accounting for 2.77% of the total regional area. The area is adjacent to the Oasis Agricultural Area at the northern foothills of the Qilian Mountains, but the current agricultural productivity and production potential of the area are significantly lower than those of the latter, and the problem of soil erosion is more prominent (Figure 7).

The northeast Tsaidam Basin is situated on the northeast edge of the Tsaidam Basin, with the Zongwu Mountain in the north. The main functions of the area are water and soil conservation. The area is 1.06 × 104 km^2^, accounting for 4.46% of the total regional area. The subdivision has similar natural conditions to Aksay (corridor region of the western Hexi Basin) and is spatially divided into different ecological restoration zones due to discontinuity. The ecological problems in this zone mainly include less droughts and sparse vegetation, which indicate a fragile natural environment, a low agricultural production potential, and the fact that agricultural and animal husbandry production operations are extensive (Figure 7).

## 5. Discussion

In previous studies, many scholars have carried out ecological vulnerability [38] and ecological security assessments [39], as well as other related studies to provide decision-making guidelines based on ecological protection and restoration by identifying important and key areas of ecological restoration [40]. The commonly used assessment models for ecosystem vulnerability studies are the pressure-state-response (PSR) [41] and the exposure-sensitive-adaptive (ESA) models [42]. Ecological security pattern studies are based on ecological networks, especially those from Europe [43] and the United States [44]. The framework of ecological security patterns comprises the “ecological sources-ecological resistance surfaces-ecological corridors” [45]. Compared with these studies, China’s ecological restoration projects emphasize the combination of important ecological areas (mountains, rivers, forests, farmlands, lakes, grasslands, etc.). Ecological restoration involves paying more attention to the systematic restoration of total regional elements to ensure the integrity of ecosystem structures and functions. China’s 13th five-year plan puts forward the concept that “mountains, rivers, forests, fields, lakes, and grasslands are a community of life” in ecological restoration. Therefore, for ecological restoration zoning, the ecosystem as a community must also be comprehensively diagnosed. Based on this, this study proposed a comprehensive diagnosis method for identifying ecological problems, and a comprehensive restoration zoning method based on different ecosystem problems, which can provide better technical support for the implementation of ecological restoration projects.

In this study, some key indicators were selected to characterize each ecosystem element. For example, the assessment of soil conservation was used to describe “mountains, water, and forests” in the ecosystem elements of the Qilian Mountains. However, there were some limitations and uncertainties. Firstly, considering the natural environmental conditions and special ecological and environmental problems in different regions, the different needs for ecological services and ecological restoration will affect the selection of various ecosystem element indicators. Therefore, the scientific index selection method for evaluating ecological restoration needs to be further explored. Secondly, the index weight assignment involved the difficulty of constructing the ERI. The application of inappropriate methods may thus have directly affected the distribution characteristics of the evaluation results and significantly increased the uncertainty. Although the method of determining index weights in this study could better reflect the extent to which various indicators involve ecological problems, the weight value will be influenced by subjective selected indicators. Further ecological restoration zoning could be studied by combining different index weight determination methods. For example, research has shown that spatial principal component analysis has advantages in ecosystem vulnerability assessment [46]. It can objectively determine the weight of the evaluation indicators and avoid subjective arbitrariness, but there is a certain problem of information loss.

When planning ecological restoration projects, the allocation of restoration areas and the cost of restoration measures are two major problems faced by decision makers, which can be further studied by considering the construction of appropriate ecological restoration development frameworks. For example, Zhang et al. [47] planned wetland restoration projects based on the framework of interval fuzzy linear programming, which can deal with the trade-off between eco-environmental benefits and economic costs. Under the background of global climate change, ecosystem restoration should not only combine the characteristics of the ecosystem itself, but also consider the impact of climate change [48]. In addition, with the development and application of remote sensing (RS), geographic information systems (GIS), global positioning systems (GPS), and other technologies, ecological restoration zoning results have become more dynamic. In future research, it is also worth paying attention to carrying out the real-time diagnosis of ecological restoration problems and make timely policy adjustments by using remote sensing image data with high spatial and temporal resolutions.

## 6. Conclusions

Based on the land use data of the resources and environmental data centers of the Chinese Academy of Sciences, the cultivated land quality data of the 1:1 million land resource map, the NDVI data of MODIS, and the big data search of the network crawler, the ecological problems related to the mountains, water, forests, fields, lakes, and grasslands of the Qilian Mountains were systematically analyzed. The study found that mining exploration and hydropower projects have been the main reasons for the ecological damage of the Qilian Mountains for nearly half a century. From 1990–2015, the land use of the Qilian Mountains changed significantly: the forestry and wetland areas diminished while the proportion of grassland areas increased. From 1990–2015, cultivated lands and built-up areas expanded significantly. The high-coverage grasslands, low-coverage grasslands, and other woodlands showed increasing trends. The shrub forestry areas, medium-coverage grasslands, sparse forests, unused lands, and water areas decreased significantly.

Here, we have presented the index of the Qilian Mountains’ ERI, from which we identified the key areas of the latter’s ecological restoration and distinguished different restoration zones. The ERIs in the southeast and southwest of the research area were relatively large, implying that an urgent restoration of ecological protection is needed in these areas. Finally, we divided the study area into three primary ecological restoration zones and eight secondary ecological restoration zones. The average restoration index of the eight ecological restoration zones was 0.61, and the partition restoration index was between 0.34 and 0.8. Of these indices, the zone with the lowest values occurred in the upper Shule River to middle Heihe River, which had the most serious comprehensive ecological problems and the highest urgency of repair. In contrast, the ERI of the Huangshui River Basin, the area with the highest ecological quality in the Qilian Mountain area, was 0.73.

Our findings can serve as a scientific basis for policy implementations for the diagnosis and restoration of ecological problems. However, there were some limitations and areas of uncertainty, such as those involving the selection of indicators and the determination of indicator weights. The scientific index selection method for evaluating ecological restoration should be further explored. Ecological restoration zoning can be studied by combining different index weight determination methods. It is also worth paying attention to carrying out real-time diagnoses of ecological restoration problems and making timely policy adjustments by combining remote sensing image data with high spatial and temporal resolutions.

## Figures and Tables

**Table 1:** Diagnosis index system of mountains, forestry, fields, lakes, and grassland ecology systems in the Qilian Mountains.

Type	Ecological Issues	Indicators	Meaning of the Index
Mountain	Soil erosion	Amount of soil conservation	Application of land use and management methods to prevent soil erosion by human or natural factors to maintain the total amount of natural soil functions
	Mining	Mine distribution density	Reference to the number of mines within a certain geographical space range
Water	Extreme precipitation	Rainstorm days	Number of days with daily precipitation exceeding 50 mm
	Uneven precipitation distribution	Annual precipitation	The sum of the average monthly precipitation in the year represents the annual precipitation
Forestry areas	Forest is degraded	Interannual change rate of forest vegetation	Changes of forest vegetation within one year
	Poor forest score quality		
Cultivated land	Low farm quality	Agricultural production potential	Agricultural production potential is the maximum possible output to be achieved annually on lands per unit of land
Grassland	Meadows are degraded	Interannual change rate of grassland vegetation	Changes of grassland vegetation within one year
People	Poverty situation	Rural per capita income	Average income of rural individuals within one year

**Table 2:** Weight of the ecological restoration evaluation index in the Qilian Mountains.

Evaluation Indicators	Ecological Restoration Object	Indicator Weight
Amount of soil conservation	Mountains; rivers; and forests	0.3
Mine distribution density	Mountains	0.1
Extreme precipitation	Rivers	0.1
Annual precipitation	Rivers	0.1
Interannual change rate of forest vegetation	Forests	0.1
Interannual change rate of grassland vegetation	Grasslands	0.1
Cultivated land grade	Cultivated lands	0.1
Rural income per capita	People	0.1

**Table 3:** Ecological restoration zones of the Qilian Mountains planning area.

First-Level Division	Secondary Division	Regional Area: Ten Thousand (km^2^)	Area Ratio (%)	Ecological Restoration Index
I—forests’ and grasslands’ water conservation areas	1—headwaters of the Datong River Basin	2.75	11.61	0.54
	2—Danghe-Dahaerteng River Basin	2.80	11.83	0.65
	5—northern confluence area of the Qinghai Lake	3.28	13.84	0.62
	6—upper Shule River to middle Heihe River	3.62	15.26	0.50
II—cultivated lands’ and grasslands ecological restoration areas	4—Oasis Agricultural Area at the northern foothills of the Qilian Mountains	3.43	14.45	0.68
	7—Huangshui Basin Valley	0.66	2.77	0.73
III—deserted grasslands’ ecological control area	3—Aksay, corridor region of the western Hexi Basin	6.11	25.78	0.56
	8—northeastern Tsaidam Basin	1.06	4.46	0.57

## Data Availability

All relevant datasets in this study are described in the manuscript.

## Appendix A

### The Specific Calculation Process of Indicators

(1) Soil Conservation

The soil conservation determination method of the Qilian Mountains adopted the general equation of soil erosion, and its calculation was performed using MATLAB. First, the potential soil erosion was calculated according to rainfall erosivity, “*R*”, soil erodibility, “*K*”, and slope length slope factor, “*LS*”. Second, under the conditions of the vegetation cover, management factor, “*C*”, and engineering measure factor, “*P*”, a value was assigned according to different land use types. Finally, the actual soil erosion was calculated by using the general soil erosion equation. The difference from the potential soil erosion was the soil conservation value. The specific formula was as follows:

First, the potential soil erosion amount was calculated. The formula was as follows [49]:

(A1) RKLS=R×K×LS

Second, in the case of vegetation cover and engineering measures, the general soil loss equation was used to calculate the actual soil loss [50]. The formula was as follows:

(A2) USLE=R×K×LS×C×P

Finally, the amount of soil conservation was defined as the difference between *RKLS* and *USLE*, expressed by the soil holding conservation (*SHC*), as follows:

(A3) SHC=RKLS−USLE

where “*R*” is the rainfall erosion force, “*K*” is the soil erodibility, “*LS*” is the long slope factor, “*C*” is the vegetation cover and management factor, and “*P*” is a project measure factor.

Rainfall erosivity, “*R*”, reflects the potential capacity of soil erosion caused by rainfall, in addition to Wischmeier’s empirical formula [51,52], which was used in the calculation:

(A4) $$R=\sum_{1}^{12}1.735\times 10^{\left[\left(1.5\times lg\frac{p_{i}^{2}}{p}\right)-0.8188\right]}$$

where “*p_i_*” is the average monthly rainfall and “*p*” is the average annual rainfall.

Soil erodibility, “*K*”, is an important index of the soil erosion equation, and the Erosion–Productivity Impact Calculator (EPIC) formula established by Williams et al. [53] was used for calculations, namely:

(A5) $$\begin{array}{ll} K= & \left\{0.2+0.3\exp\left[-0.025san\left(1-\frac{sil}{100}\right)\right]\right\}\times {\left[\frac{sil}{cla+sil}\right]}^{0.3}\times \\ & \left[1-0.025\frac{c}{c+\exp\left(3.72-2.95c\right)}\right]\times \left[1-0.7\frac{sn1}{sn1+\exp\left(22.9sn1-5.51\right)}\right] \end{array}$$

where “*san*”, “*sil*”, “*cla*”, and “*c*” represent the contents of sand, silt, clay particles, and organic carbon in the soil (%), respectively, and “*snl*” is equal to 1 − *san*/100.

The topographic factor “*LS*” reflects the relationship between slope and surface conditions, and is the distance from which rain drops or sediments flow until the energy disappears, reflecting the impact of the topographic and geomorphological characteristics on soil erosion. For different slopes, the model can automatically calculate the value of the terrain factor and set the slope threshold to 25%, depending on the actual situation. The surface cover management, factor “*C*”, refers to the ratio of the soil loss of a specific crop or natural vegetation under the same soil, terrain, and rainfall, mainly affected by soil water, early land use mode, and vegetation crops’ planting sequence to reflect the impact of vegetation or crops and management measures on soil loss. This value is between 0–1. The soil conservation measure, factor “*P*”, indicates the ratio of soil loss to soil loss after slope planting, reflecting the difference in soil loss caused by the differences in vegetation management measures. This is one of the most important factors to inhibit soil erosion, whose range is between 0–1. “*P*” and “*C*” can be obtained from the relevant literature [54].

(2) Mine Distribution Acquisition

The mining strength was quantitatively analyzed according to the mine distribution density (MD). Mine distribution data were obtained using mine information searching technologies. Mine information searching was mainly based on the Amap application programming interface (API), and was completed through secondary development. In other words, relevant means such as a point of interest (POI) search, geographic position inverse queries, and so on, were used to obtain the longitude and latitude coordinates of the mines, and then GIS software was used to generate the spatial distribution of the mines. This research used a Python programming crawler program to access the Amap WEB service, search for place names related to “mines”, and determine the density of the Qilian Mountain mines based on their geographical location.

(3) Precipitation and Extreme Precipitation

The level of precipitation in China was mainly based on daily precipitation values. In Northern China, the standard of daily precipitation is 10 mm/d or less for light rain, 10–25 mm/d for moderate rain, 25–50 mm/d for heavy rain, and 50 mm/d and up for extreme precipitation. Therefore, this study selected days with daily precipitation values greater than 50 mm as the extreme precipitation index (rainstorms). In this paper, the daily precipitation and radiation data with a resolution of 250 m from 2001–2015 were obtained from the daily dataset of China’s surface climate data (V3.0), and the spatial distribution was obtained by interpolation.

(4) Interannual Change Rate of Forests and Grassland Vegetation

We evaluated the 16-day synthetic normalized difference vegetation index (NDVI) products (MYD13A2 and MOD13A2) of grassland vegetation based on the 1000 MODIS for the years 2001–2015. To obtain the vegetation NDVI time series curve of the Qilian Mountains from 2001–2015, the noise time series curve of “cloud pollution” with quality control documents was eliminated, and the NDVI time series curve of each pixel was reconstructed with a Savitzky–Golay filter.

The change in vegetation in the Qilian Mountains was characterized by the interannual change rate of vegetation’s NDVI, which was calculated using the slope of the trend line in linear regression analyses. The calculation formula was as follows [55,56,57]:

(A6) $$\theta_{slope}=\frac{n\times \sum_{i=1}^{n}i\times NDVI_{i}-\sum_{i=1}^{n}i\times \sum_{i=1}^{n}NDVI_{i}}{n\times \sum_{i=1}^{n}i^{2}-{\left(\sum_{i=1}^{n}i\right)}^{2}}$$

where “*θ_slope_*” is the slope of the fitted trend line, “*n*” is the study period, and “*NDVI*” is the vegetation’s *NDVI* for the *i*-th year. When *θ_slope_* > 0, there was a downward trend in “*NDVI*” in the time series of the pixel; otherwise, “*NDVI*” had an upward trend.

## References

[1] Milan R. (1995). The ecological and environmental education. Ekol. Bratisl..

[2] Fischer S., Thatje S. (2016). Temperature effects on life-history traits cause challenges to the management of brachyuran crab fisheries in the Humboldt Current: A review. Fish. Res..

[3] Omernik J.M., Griffith G.E. (2014). Ecoregions of the Conterminous United States: Evolution of a Hierarchical Spatial Framework. Environ. Manag..

[4] Olson D.M., Dinerstein E. (1998). The Global 200: A Representation Approach to Conserving the Earth’s Most Biologically Valuable Ecoregions. Conserv. Biol..

[5] Omernik J.M. (1995). Ecoregions: A framework for managing ecosystems. Georg. Wright Forum.

[6] Box W.E. (1976). Global classification of natural terrestrial ecosystems. Vegetatio.

[7] Bailey R.G., Zoltai S.C., Wiken E.B. (1985). Ecological regionalization in Canada and the United States. Geoforum.

[8] Zogaris S., Economou A.N., Dimopoulos P. (2009). Ecoregions in the Southern Balkans: Should Their Boundaries Be Revised?. Environ. Manag..

[9] Zhou L., Sun D., Xu J. (2015). Zoning assessment of water environmental supporting capacity for socioeconomic development in the Huaihe River Basin, China. J. Geogr. Sci..

[10] Yang S.Y., Tang T., Cai Q.H., Xiao W., Wang X.Z., Li F.Q., Tang J. (2012). Aquatic eco-regionalization of Erhai Lake Basin, Yunnan Province of Southwest China. Chin. J. Ecol..

[11] Liu X., Liu L., Peng Y. (2017). Ecological zoning for regional sustainable development using an integrated modeling approach in the Bohai Rim, China. Ecol. Modell..

[12] Deboudt P., Dauvin J.-C., Lozachmeur O. (2008). Recent developments in coastal zone management in France: The transition towards integrated coastal zone management (1973–2007). Ocean Coast. Manag..

[13] Pikitch E.K., Santora C., Babcock E.A., Bakun A., Bonfil R., Conover D.O., Dayton P., Doukakis P., Fluharty D., Heneman B. (2004). Sainsbury. Ecosystem-Based Fishery Management. Science.

[14] Li F., Xu M., Liu Q., Wang Z., Xu W. (2014). Ecological restoration zoning for a marine protected area: A case study of Haizhouwan National Marine Park, China. Ocean Coast. Manag..

[15] Yang X., Zhang F., Luo C., Zhang A. (2019). Farmland Ecological Compensation Zoning and Horizontal Fiscal Payment Mechanism in Wuhan Agglomeration, China, From the Perspective of Ecological Footprint. Sustainability.

[16] Wang G., Yang D., Xia F., Zhong R., Xiong C. (2019). Three Types of Spatial Function Zoning in Key Ecological Function Areas Based on Ecological and Economic Coordinated Development: A Case Study of Tacheng Basin, China. Chin. Geogr. Sci..

[17] Wang Y., Li H., Ren J. (2019). Delimitation and Zoning of Natural Ecological Spatial Boundary Based on GIS. Asian J. Agric. Res..

[18] Mamat K., Du P., Ding J. (2017). Ecological function regionalization of cultural heritage sites in Turpan, China, based on GIS. Arab. J. Geosci..

[19] Wang L., Wang W., Yang X. Eco-Environmental Zoning: A GIS-Based Approach. Proceedings of the 2008 2nd International Conference on Bioinformatics and Biomedical Engineering.

[20] Shao J., Yang L., Peng L., Chi T., Wang X. (2015). An Improved Artificial Bee Colony-Based Approach for Zoning Protected Ecological Areas. PLoS ONE.

[21] Read A.D., West R.J. (2010). Qualitative risk assessment of multiple-use marine park effectiveness–A case study from NSW, Australia. Ocean Coast. Manag..

[22] Guo Y. (2019). Spatial Distribution and Simulation of Cropland Abandonment in Wushan County, Chongqing, China. Sustainability.

[23] Liu Y., Song W., Deng X. (2019). Understanding the spatiotemporal variation of urban land expansion in oasis cities by integrating remote sensing and multi-dimensional DPSIR-based indicators. Ecol. Indic..

[24] Abutaleb K.A.A., Asmaa M., Hassan E., Ahmed M. (2018). Climate Change Impacts, Vulnerabilities and Adaption Measures for Egypt’s Nile Delta. Earth Syst. Environ..

[25] Yulin S. (1990). 1:1 Million Land Resources Map of China.

[26] Alcantara C., Kuemmerle T., Prishchepov A.V., Radeloff V.C. (2012). Mapping abandoned agriculture with multi-temporal MODIS satellite data. Remote Sens. Environ..

[27] Qian X., Zhu H. (2018). A 1-km grid dataset of industrial output value in China (2010). China Sci. Date.

[28] Xiaoming W., Junxing Y., Wei S. (2018). Pollution status of agricultural land in China: Impact of land use and geographical position. Soil Water Res..

[29] Bliss S. (2011). United Nations International Year of Forests 2011. Geogr. Bull..

[30] Wang P., Zhang L., Li Y., Jiao L., Wang H., Yan J., Lü Y., Fu B. (2017). Spatio-temporal characteristics of the trade-off and synergy relationships among multiple ecosystem services in the Upper Reaches of Hanjiang River Basin. Acta Geogr. Sin..

[31] Gao Y., Liu H., Liu G. (2017). The spatial distribution and accumulation characteristics of heavy metals in steppe soils around three mining areas in Xilinhot in Inner Mongolia, China. Environ. Sci. Pollut. Res..

[32] Li H., Song W. (2020). Characteristics of Climate Change in the Lancang-Mekong Sub-Region. Climate.

[33] Han Z., Song W., Deng X., Xu X. (2018). Grassland ecosystem responses to climate change and human activities within the Three-River Headwaters region of China. Sci. Rep..

[34] Deng X., Huang J., Rozelle S., Uchida E. (2006). Cultivated land conversion and potential agricultural productivity in China. Land Use Policy.

[35] Jian S., Deng G. (2014). The Chinese Urban and Rural per Capita Income and Trend Analysis. Appl. Math..

[36] Ding L., Zhao W., Huang Y., Cheng S., Liu C. (2015). Research on the Coupling Coordination Relationship between Urbanization and the Air Environment: A Case Study of the Area of Wuhan. Atmosphere.

[37] Liao C., Yue Y., Wang K., Fensholt R., Tong X., Brandt M. (2018). Ecological restoration enhances ecosystem health in the karst regions of southwest China. Ecol. Indic..

[38] Li H., Song W. (2021). Spatiotemporal Distribution and Influencing Factors of Ecosystem Vulnerability on Qinghai-Tibet Plateau. Int. J. Environ. Res. Public Health.

[39] Su X., Zhou Y., Li Q. (2021). Designing Ecological Security Patterns Based on the Framework of Ecological Quality and Ecological Sensitivity: A Case Study of Jianghan Plain, China. Int. J. Environ. Res. Public Health.

[40] Kang J., Zhang X., Zhu X., Zhang B. (2021). Ecological security pattern: A new idea for balancing regional development and ecological protection. A case study of the Jiaodong Peninsula, China. Glob. Ecol. Conserv..

[41] Jin X., Jin Y., Mao X. (2019). Ecological risk assessment of cities on the Tibetan Plateau based on land use/land cover changes—Case study of Delingha City. Ecol. Indic..

[42] Dieleman H. (2017). Urban agriculture in Mexico City; balancing between ecological, economic, social and symbolic value. J. Clean. Prod..

[43] Jongman R.H.G. (1995). Nature conservation planning in Europe: Developing ecological networks. Landsc. Urban Plan..

[44] Fábos J.G. (2004). Greenway planning in the United States: Its origins and recent case studies. Landsc. Urban Plan..

[45] Peng J., Zhao H., Liu Y. (2017). Urban ecological corridors construction: A review. Acta Ecol. Sin..

[46] Xue L., Wang J., Zhang L., Wei G., Zhu B. (2018). Spatiotemporal analysis of ecological vulnerability and management in the Tarim River Basin, China. Sci. Total Environ..

[47] Zhang Y., Shen J. (2021). Wetland Restoration Planning Approach Based on Interval Fuzzy Linear Programming under Uncertainty. Int. J. Environ. Res. Public Health.

[48] Jiang C., Wang F., Zhang H., Dong X. (2016). Quantifying changes in multiple ecosystem services during 2000–2012 on the Loess Plateau, China, as a result of climate variability and ecological restoration. Ecol. Eng..

[49] Huber S., Prokop G., Arrouays D., Banko G., Bispo A., Jones R.J.A., Kibblewhite M.G., Lexer W., Moller A., Rickson R.J. (2008). Environmental Assessment of Soil for Monitoring: Volume I Indicators & Criteria.

[50] Wischmeier W.H., Smith D.D. (1978). Predicting Rainfall Erosion Losses—A Guide to Conservation Planning.

[51] Silva A.M.D. (2004). Rainfall erosivity map for Brazil. Catena.

[52] Renard K.G., Foster G.R., Weesies G.A., Mccool D.K., Yoder D.C. (1997). Predicting Soil Erosion by Water: A Guide to Conservation Planning with the Revised Universal Soil Loss Equation (RUSLE).

[53] Williams J.R., Arnold J.G. (1997). A system of erosion—Sediment yield models. Soil Technol..

[54] Li S., Wang Z., Zhang Y. (2017). Crop cover reconstruction and its effects on sediment retention in the Tibetan Plateau for 1900–2000. J. Geogr. Sci..

[55] Ma M., Frank V. (2006). Interannual variability of vegetation cover in the Chinese Heihe River Basin and its relation to meteorological parameters. Int. J. Remote Sens..

[56] Piao S., Wang X., Ciais P., Zhu B., Wang T., Liu J. (2011). Changes in satellite-derived vegetation growth trend in temperate and boreal Eurasia from 1982 to 2006. Glob. Chang. Biol..

[57] Xia H., Li A., Feng G., Li Y., Qin Y., Lei G., Cui Y. (2018). The Effects of Asymmetric Diurnal Warming on Vegetation Growth of the Tibetan Plateau over the Past Three Decades. Sustainability.

